# Wood Usage and Fire Veneration in the Pamir, Xinjiang, 2500 yr BP

**DOI:** 10.1371/journal.pone.0134847

**Published:** 2015-08-26

**Authors:** Hui Shen, Xinhua Wu, Zihua Tang, Xinying Zhou, Nan Sun, Xiaoqiang Li

**Affiliations:** 1 Key Laboratory of Vertebrate Evolution and Human Origin of Chinese Academy of Sciences, Institute of Vertebrate Paleontology and Paleoanthropology, Chinese Academy of Sciences, Beijing, China; 2 Department of Earth Science, University of Chinese Academy of Sciences, Beijing, China; 3 The Institute of Archaeology, Chinese Academy of Social Sciences, Beijing, China; 4 Key laboratory of Cenozoic Geology and Environment, Institute of Geology and Geophysics, Chinese Academy of Sciences, Beijing, China; 5 Department of earth science and resources, Chang’an University, Shaanxi, China; Institute of Tibetan Plateau Research, CHINA

## Abstract

Located on the Pamir Plateau in Xinjiang Province, China, the Ji’erzankale Necropolis dates back to 2500 yr BP. Many materials that have been unearthed in this cemetery, including shoo konghou (musical instrument), bronze mirrors and glass beads, suggest cultural transference between East and West. Furthermore, small-sized and rounded fire altars made from sweet-scented *Sabina* were found for the first time and regarded as implements for fire veneration. We identified 70 wooden objects from 25 tombs within the Necropolis, and found that each object had been made from one of seven tree species. Analysis revealed that the inhabitants of the region mainly used the most widely available types of wood, namely *Betula* and *Populus*. People also specifically chose inflammable *Populus* wood to make hearth boards and hand drills (both are used for making fire by drilling), rigid *Betula* wood to craft wooden plates. *Salix *was used for fashioning wooden sticks, while sweet-scented *Sabina* was the preferred choice for making fire altars. *Lonicera* was selected for arrow shaft manufacture and *Fraxinus syriaca*, which has a beautiful grain, was chosen for making musical instruments. Conscious selection of different types of wood indicates that people of the Pamir Plateau were aware of the properties of various types of timbers, and were able to exploit these properties to the full. In turn, this demonstrates their wisdom and their ability to survive in, and adapt to, their local environment.

## Introduction

The Pamir Plateau is situated in an arid region of central Asia. Since the late Stone Age, this area has been a cultural crossroad [1,2]. Around 3500 yr BP, the Pamir was influenced by the Andronovo culture which was then widespread in Siberia and central Asia. After that, a series of exotic religions, including Zoroastrianism, Buddhism, Nestorianism and Islam, spread to this region [3,4]. Furthermore, opening of the southern route of the Silk Road led to more frequent and varied cultural exchanges. Merchants, tourists, monks, and ambassadors from various civilizations all introduced to the Pamir their own cultures, which later spread to, interacted with and integrated into central China to the east, Mesopotamia to the west, the Indus Valley to the south and Semiryechye to the north [5]. Therefore, this region is an ideal place for research into cultural transmission.

According to stone inscriptions from Naqsh-e Rustam in Persia, the majority of the Pamir population 2000 years ago belonged to the Saka [6], a nomadic people who left little in the way of a permanent cultural layer due to their itinerant lifestyle. Their tombs therefore provide vital clues regarding their social life, culture, religion and environmental adaptability [6]. However, knowledge of these questions remains sketchy and reliant on secondary historical documents because of the paucity of relevant archaeological sites [6,7]. The Ji’erzankale Necropolis in Tashkurgan County is located in the interior of the Pamir Plateau, and many objects have been unearthed from this site. Among these remains, wooden objects provided direct evidence of palaeovegetation and resource exploitation.

In addition, fire altars with rounded shape were unearthed for the first time [3]. Black combustion marks were clear in the inwall and inside of it, 15 stones with black burning traces on the surface were placed [3]. This burning feature showed that they were on fire when buried near the dead and thus provided important clues bearing on early forms of burial custom and belief [3]. Abundant bamboo combs, glass beads, and tin and bronze mirrors shed light on the intercultural exchanges that were characteristic of this region.

Early peoples were invariably dependent for their survival on the local environment, and especially on the use of timber. Evidence of timber use is often well-preserved in archaeological sites [8–10]. Under conditions of incomplete burning, and/or in dry or waterlogged environments in which microorganic activity is weak, wood can keep its anatomical structure [11,12]. Integrated identification and study of wood and charcoal found in archaeological sites started at the end of the last century. As a primary plant record, preserved wood provides important clues that are helpful in reconstructing palaeovegetation and revealing how local residents used wood and interacted with their local environment [13–15].

In this paper, we studied a total of 70 wood samples unearthed from the Ji’erzankale Necropolis, identifying them taxonomically and analyzing their properties in order to reveal the burial customs and patterns of plant utilization of the people lived in Pamir Plateau 2500 yr BP. We also attempted to shed light on the cultural communications between East and West at that time.

## Materials and Methods

In 2013 and 2014, Ji’erzankale Necropolis was unearthed by the Xinjiang Archaeological Team of the Chinese Academy of Social Sciences directed by Prof. Wu (the second author of this paper). This project was authorized by the State Administration of Cultural Heritage with archaeological excavation license number 349 (2014). In regard to sampling method, we took a small piece of sample from each woodware for identifying, avoiding damaging to these objects. Subsequently, boiling test was used for some rigid samples for softening purpose, with permission of the Xinjiang Archaeological Team of the Chinese Academy of Social Sciences.

**Fig 1:**
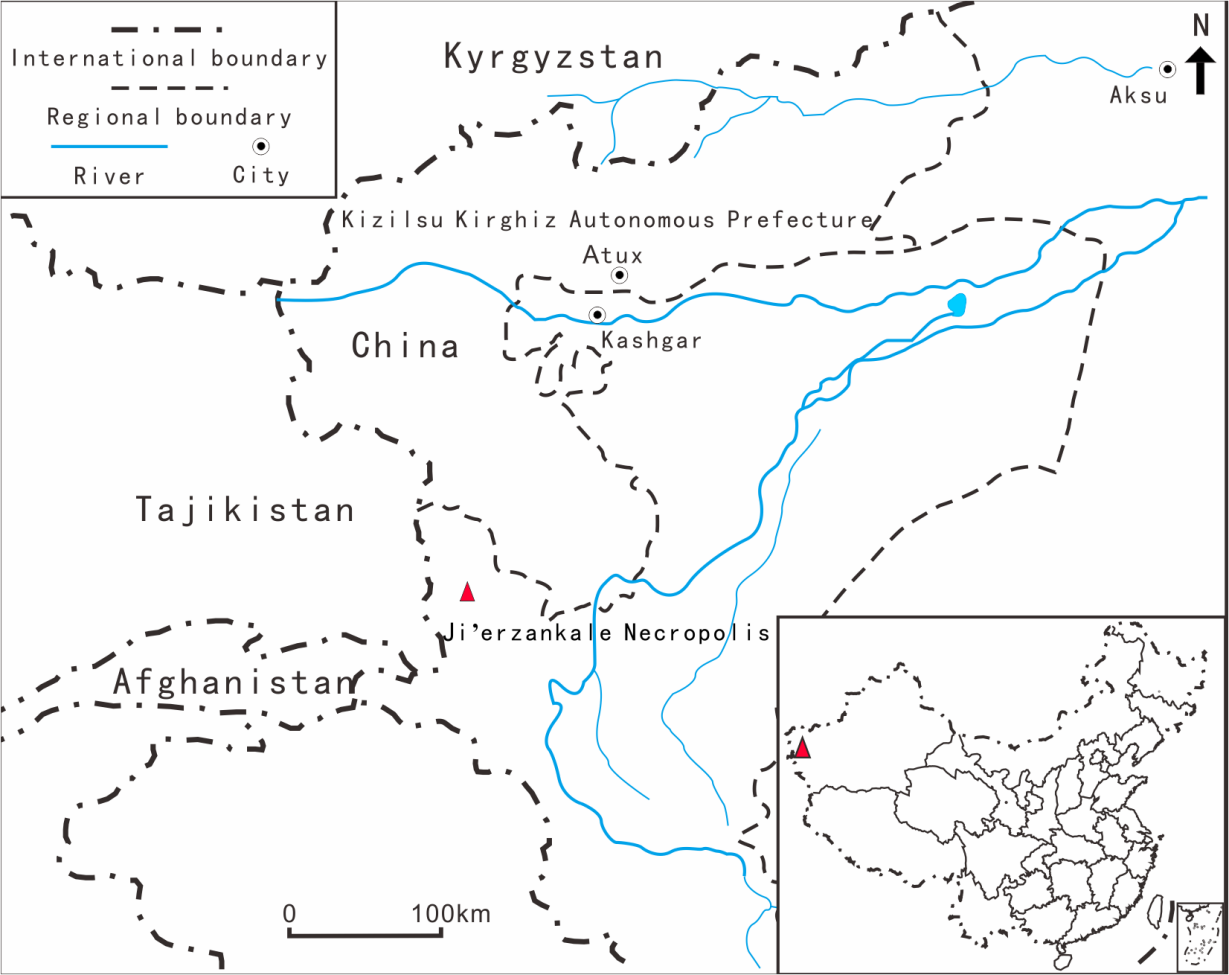
Location of Ji’erzankale Necropolis. The red triangle represents the Ji’erzankale Necropolis site.

The Ji’erzankale Necropolis is located in Qushiman Village, Tashkurgan Tajik Autonomous County, Xinjiang (75°12'11", 37°50'54") (Fig 1). This region lies in the southwestern part of Xinjiang, east Pamir Plateau, north of the Karakoram, and west of the Tarim Basin. The mean annual temperature is 3.6°C, mean annual precipitation is <70mm, and mean annual evaporation can reach 2300mm. It is thus a cold, dry, high-radiation climate [16].

**Fig 2:**
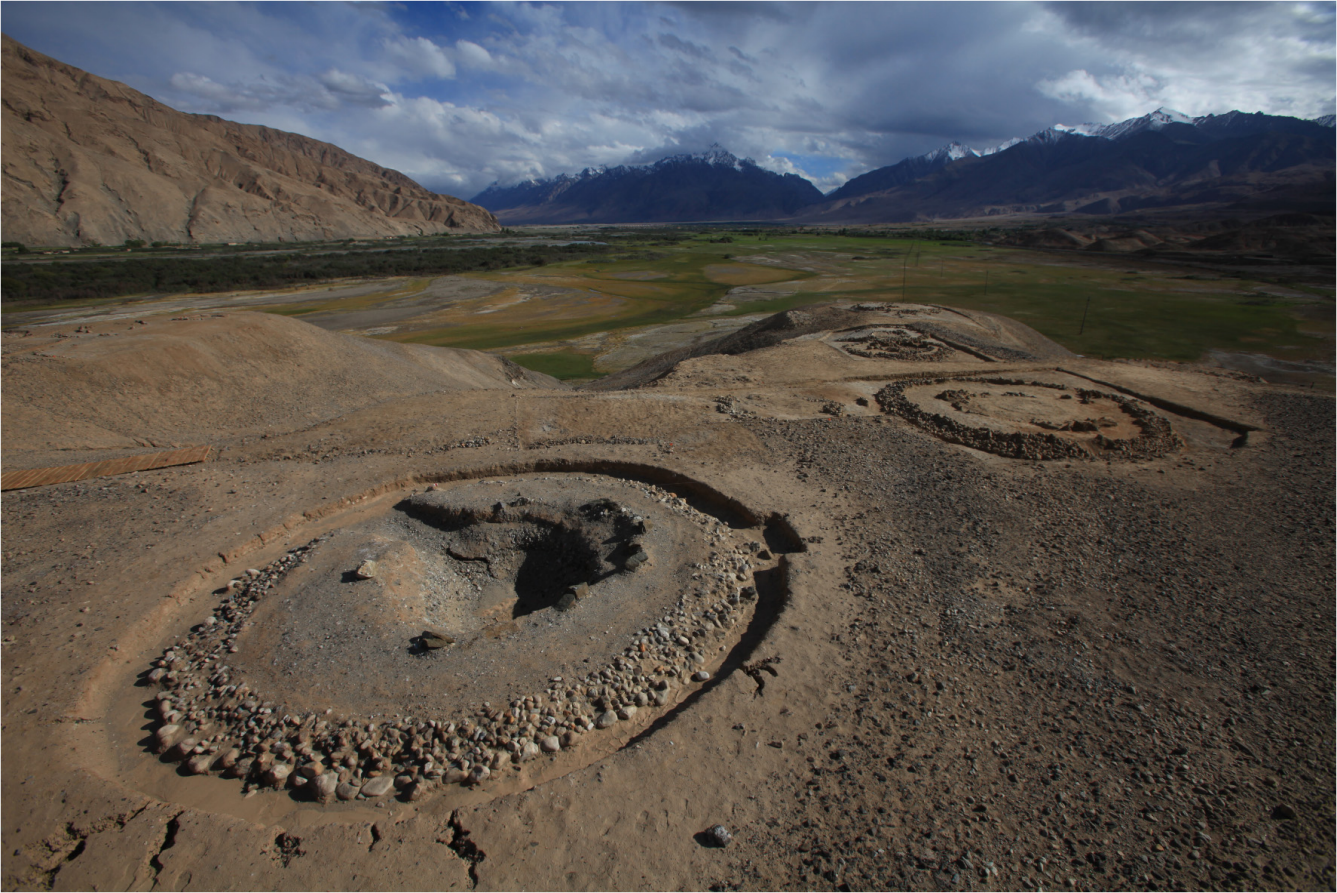
Photograph of Ji’erzankale Necropolis. Tombs were surrounded by stone circles and a large area of black and white stones was laid on the surface.

The cemetery was divided into districts A, B and C, containing 50 tombs numbered M1-M50 in total (7 in District A, 34 in District B and 9 in District C). A large area of black and white stones arranged in long strip shape was laid on the surface (Fig 2). All tombs were pit graves and built on bedrock or gravel layer in flood plain. On the basis of human bone study, both primary and secondary burial existed [3]. Apart from graves with human corpse, some tombs like M4 were only buried with hawk cranium [3]. Abundant pottery items, stone tools, copper objects, ironware, wooden items, bone objects, glass beads, and a variety of other artefacts were found during this process [3]. AMS ^14^C Beta Analytic dating of two wood samples, representing an arrow shaft from M14 and a fire altar from M15, yielded respective dates of 2750–2710 yr BP and 2540–2355 yr BP showed in Table 1. The dates show that the Ji’erzankale Necropolis is about 2500 years old.

**Table 1:** Accelerator mass spectrometry (AMS) radiocarbon dates from the Ji’erzankale Necropolis.

Tomb.no	use	Lab.no	Sample type	δ^13^C	AMS age(BP)	Calibrated age (cal BP,2σ)
**M14**	arrow shaft	Beta-400296	wood	-21.7	2570±30	2750–2700
**M15**	fire altar	Beta-400297	wood	-22.3	2430±30	2540–2355

**Fig 3:**
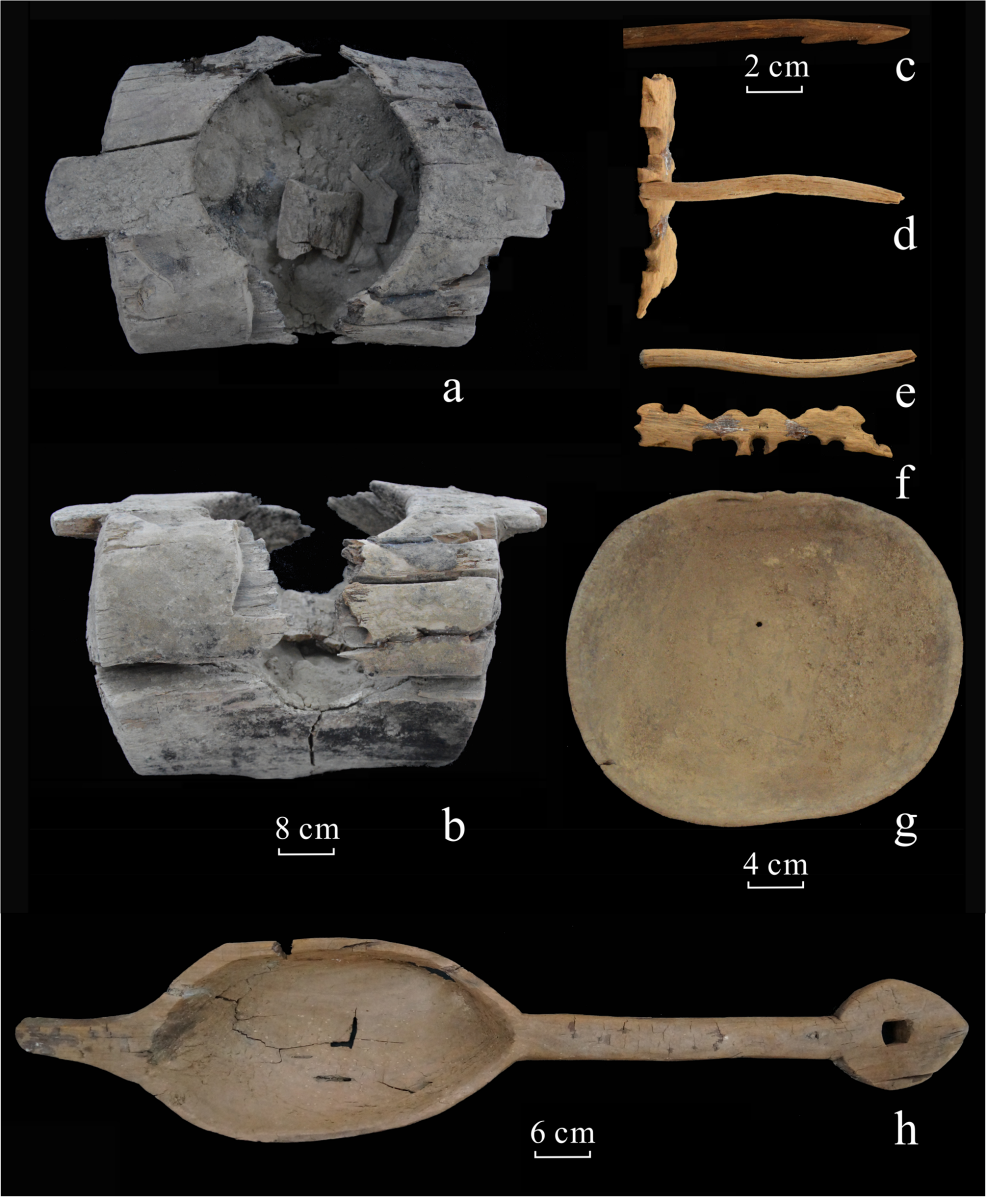
Photographs of wooden objects from Ji’erzankale Necropolis. (a)Top view of fire altar; (b) Front view of fire altar; (c) Arrow shaft; (d) Hearth board and hand drill; (e) Hand drill; (f) Hearth board; (g) Top view of wooden plate; (h) Top view of shoo konghou.

For this paper, we studied a total of 70 selected wooden objects from 25 tombs within the Necropolis, including 11 fire altars (Fig 3A and 3B), 8 arrow shafts (Fig 3C), 4 hand drills (Fig 3D and 3E), 3 hearth boards (Fig 3D and 3F), 3 wooden sticks, 30 wooden plates (Fig 3G), 6 woodwares, 2 pieces of shoo konghou (Fig 3H), 1 harp, 1 crutch and 1 wooden bowl (full details in S1 Table). About a volume of 4 cm^3^ samples were taken from each wood object for identification. Because some samples were highly rigid, hydrothermal softening was used in the laboratory tests [17]. First, we put them into beakers full of boiled water, and applied heat continuously for 24 hours. Second, we cut a transverse section through the softened wood, after drying, and examined the section through a microscope in order to arrive at a preliminary identification for the sample. Third, three sections (transverse, radial and tangential) were handmade and then metal-sprayed to allow clear images to be captured using a scanning electron microscope (ZEISS MA EVO25). Fourth, we identified the species of wood, based on the arrangement, shape and size of the pipes or tracheids, the presence or absence of resin canal in the transverse section, the height and width of woodray on the surface of the tangential section, the arrangement of the pits in the walls of the pipes or tracheids, and the pattern of ray parenchyma cells and cross-field pitting in the joint ray and tracheid cell walls in the radial section. Identifications were made using standard reference works [18–21].

## Results

The 70 objects considered in this study were categorized with respect to both object type and the taxonomic identity showed in Table 2. All taxonomic identifications are based on anatomical features visible in the transverse, radial and tangential sections. Four samples, representing two wooden plates from tombs M22 and M24, one wooden bowl from M33, and one fire altar from M35, could not be identified because of deformation of the wood-ray parenchyma exposed in the transverse section.

**Table 2:** The use and species of wood found in Ji’erzankale Necropolis.

	*Sabina* sp.	*Populus* sp.	*Betula* sp.	*Salix* sp.	*Lonicera* sp.	*Elaeagnus angustifolia*	*Fraxinus syriaca*	unidentified
**fire altar**	3	1	6					1
**wooden plate**		8	19	1				2
**hearth board**	1	2						
**hand drill**		2		2				
**arrow shaft**					8			
**wooden stick**				3				
**woodware**		1	3	1		1		
**shoo konghou**		1					1	
**harp**							1	
**crutch**				1				
**wooden bowl**								1
**total**	4	15	28	8	8	1	2	4

### *Sabina* sp.

**Fig 4:**
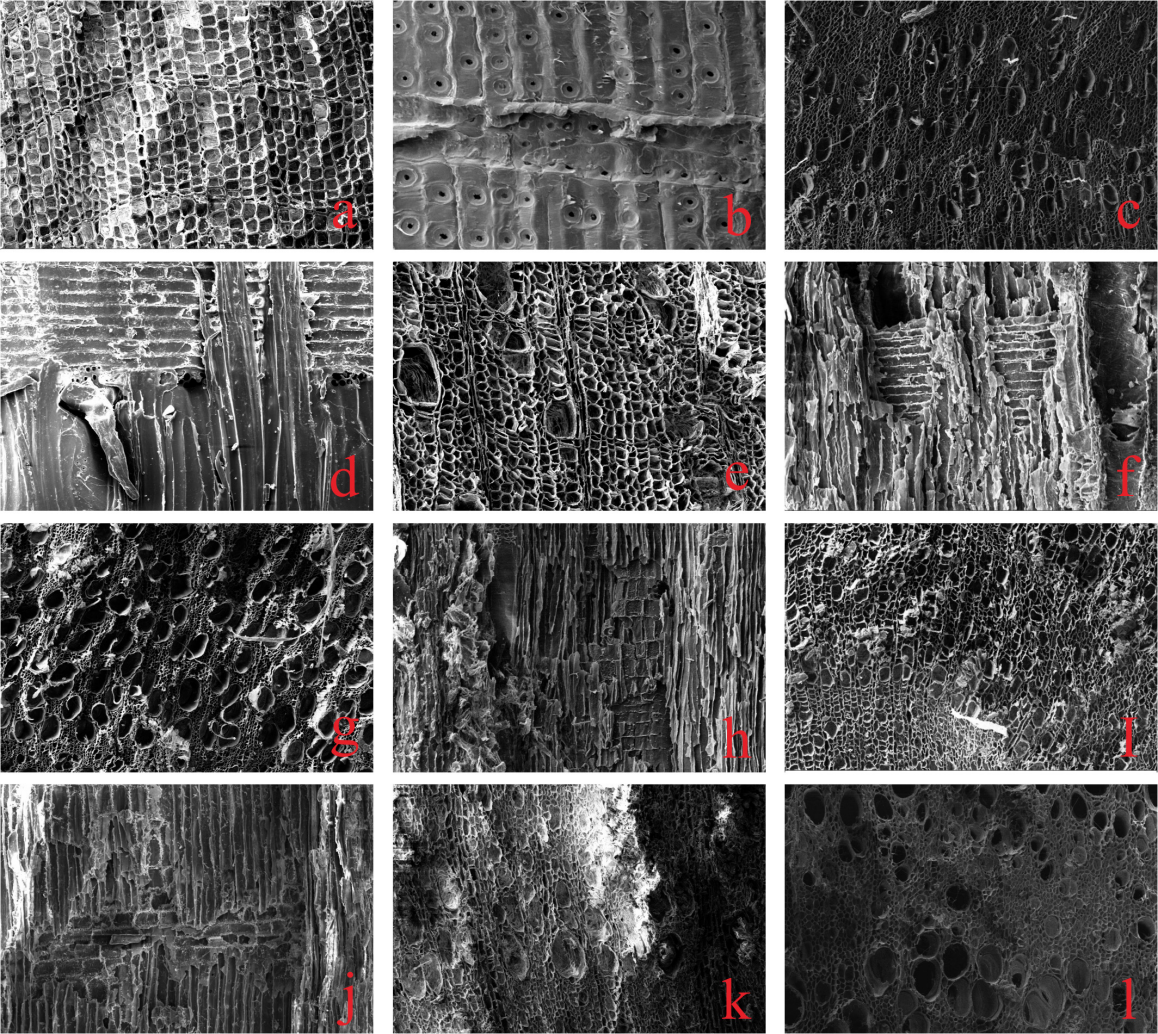
Microphotographs of archaeological wood from Ji’erzankale Necropolis. (a) *Sabina*, transverse section; (b) *Sabina*, radial section; (c) *Populus*, transverse section; (d) *Populus*, radial section; (e) *Betula*, transverse section; (f) *Betula*, radial section; (g) *Salix*, transverse section; (h) *Salix*, radial section; (i) *Lonicera*, transverse section; (j) *Lonicera*, radial section; (k) *Elaeagnus angustifolia*, transverse section; (l) *Fraxinus syriaca*, transverse section.

In transverse section, pores in early wood are rounded, quadrate and polygonal, whereas pores in late wood are oblong and polygonal. The transition from early wood to late wood is gradual. Pits are single-row and rounded or oval. Cross-field pits are cupressoid. Pore walls have warty layers. Thread-thickening is absent. Rays are uniseriate and approximately 10 cells in height. Resin canals are absent (Fig 4A and 4B).

### *Populus* sp.

Growth rings are moderately distinct, and the transition from early wood to late wood is gradual. Vessels are rounded or oval in transverse section and mostly occur in radial multiples of 2–5, with a either clustered or solitary. Perforations are simple in slight-oblique end walls. Inter-vessel pits are rounded or oval, and included. Axial parenchyma is rare. Most of the rays are uniseriate, and the majority of them are 10–18 cells in height. Spiral thickening is absent (Fig 4C and 4D).

### *Betula* sp.

Growth rings are distinct and the wood is diffuse porous. Pores are mostly solitary, but a few occur in radial multiples or clusters. Vessels are rounded or oval in transverse section. Perforations are scalar form and oblique. Inter-vessel pits are oval. Radial parenchyma is rare or terminal. Wood rays are uniseriate with a height of 3–10 cells, or multiseriate with a width of 2–3 cells and a height of about 20 cells. Heterocellular and homocellular rays coexist (Fig 4E and 4F).

### *Salix* sp.

Growth rings are moderately distinct and the wood is diffused porous. Pores are mostly solitary, but a few occur in radial multiples or clusters. Vessels are oval or elliptical in transverse section. Perforations are simple in slight-oblique to oblique end walls, and inter-vessel pits are alternate and mainly polygonal. Axial parenchyma is rare. Rays are heterocellular and uniseriate, with a height of about 10 cells. Spiral thickening is absent (Fig 4G and 4H).

### *Lonicera* sp.

Growth rings are distinct. Vessels in transverse sections are diffuse and pores are mostly solitary, with a few in radial multiples of 2–3. Perforations are mostly simple in very oblique end walls. Inter-vessel pits are alternate to opposite, and are rounded, polygonal or oblong. Rays are 1-3(4) seriate, with a height of about 10–20 cells. Spiral thickening appears on the wall of vessels (Fig 4I and 4J).

### Elaegnus angustifocia

Growth rings are distinct. Early wood vessels are solitary, in tangential to radial multiples of 2–4, or in small clusters, and are rounded in transverse section. Late wood vessels, by contrast, are rounded to angular. Perforations are simple in oblique to transverse end walls. Rays are homocellular, and composed of procumbent cells. Uniseriate rays are usually 2–10 cells high, and multiseriate rays are 2–5 cells wide and up to 35(58) cells high. Crystal is not observed (Fig 4K).

### Fraxinus syriaca

Growth rings are distinct. Vessels are usually solitary, in radial multiples of 2-3(4) or rarely in small clusters. The vessels are rounded in cross-section. Perforations are simple in horizontal to oblique end walls. Parenchyma is vasicentric in the early wood, but aliform and confluent in the end of growth ring. Rays are homocellular, 1–4 cells wide, and usually 3–10 cells high. Crystal is not observed (Fig 4L).

**Fig 5:**
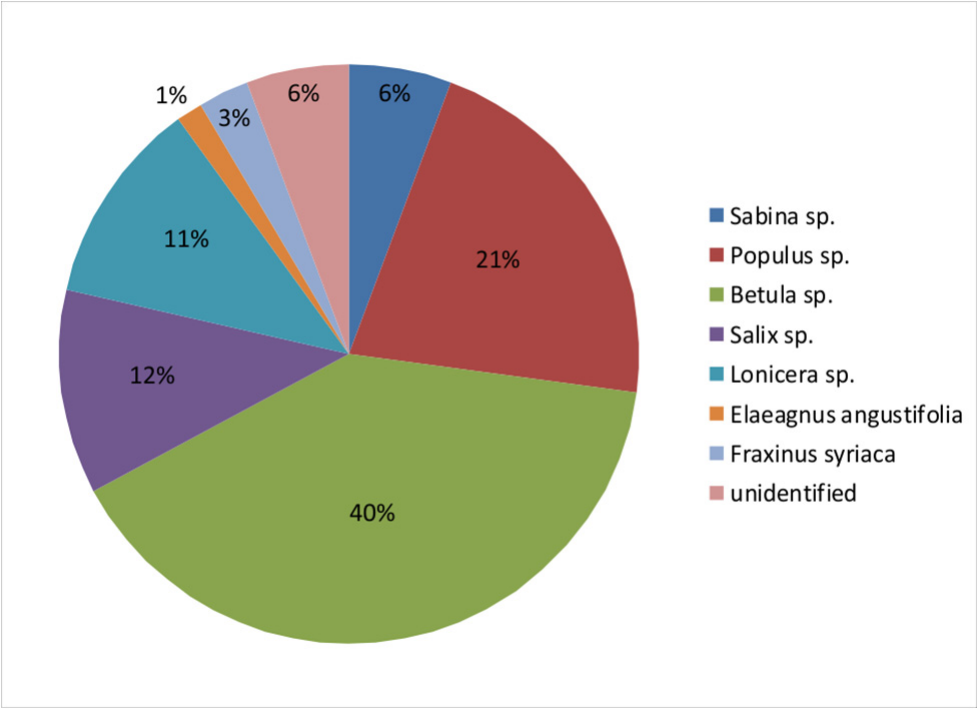
Percentage of different wood species from Ji’erzankale Necropolis. *Betula* sp. is the most species with percentage of 40%, while *Populus* sp. (21%), *Salix* sp. (11%), *Lonicera* sp. (11%), unidentified (7%), *Sabine* sp. (6%), *Fraxinus syriaca* (3%), *Elaeagnus angustifol* (1%).

Looking at our 70 wooden remains, we found that a total of seven species (28 *Betula *sp., 15 *Populus* sp., 8 *Salix* sp., 8 *Lonicera* sp., *4 Sabina* sp., 2 *Fraxinus syriaca*, and 1 *Elaegnus angustifocia*) were selected and utilized by the early residents of the Pamir Plateau. The timber from different trees was worked in different ways and for a range of purposes, including the production of fire altars for fire veneration, wooden plates for holding produce, and wood sticks and arrow shafts for protection. Among the selected trees, *Betula* and *Populus* were widely used, wood from one of these taxa having been used to manufacture 61% of the objects. *Sabina*, *Fraxinus syriaca*, and *Elaegnus angustifocia* were used less often, and account for a total of 10% of the objects. *Salix* and *Lonicera*, were each used to produce 11% of the objects (Fig 5). For individual tombs, we analyzed the tombs M14, M23 and M25 as these three tombs yielded the most wooden objects. Eleven objects made from four species of wood (in this case *Betula*, *Populus*, *Salix*, and *Lonicera*) were found in M14, while six objects made from two species were found in M23 and six objects made from four species were found in M25. This analysis revealed that people used a wide range of available local resources, rather than just selecting one kind of timber. *Vis-à-vis* the different timbers used to manufacture objects of specific types, the fire altars investigated in the analysis were made from *Betula*, *Populus*, or *Sabina*, with one fire altar made from unidentified wood. Wooden plates were made from *Betula*, *Populus* or *Salix*, with two from unidentified wood, and hearth boards were crafted from *Sabina* or *Populus*. Hand drills were made from *Populus* or *Salix*. All eight arrow shafts were from *Lonicera*, whereas the three wooden sticks and one crutch were manufactured from *Salix*. The three musical instruments were made from *Fraxinus syriaca* or *Populus*.

## Discussion

The wooden remains unearthed in archaeological sites provide direct proof of the utilization of different timber types by ancient peoples. They also shed light upon tribal, cultural, religious and human activity, and provide evidence of ancient humans’ adaptation to, and influence on, the local environment [22,23]. In addition, wooden remains play a vital role in the reconstruction of fire [14,15,24], vegetation[8,9,25] and palaeoclimate histories [13,26].

Obviously, fire use is inseparable from a dependence on local timber. The criteria governing the selection by indigenous peoples of particular wood types have long been debated, and can be summarized thus: 1) function, *e*.*g*. pine was widely used for funerary pyre in three prehistoric necropolises from Poland, possible in part because pine can release a turpentine perfume while burning [27]; 2) availability, as more widely-distributed species will clearly be selected and used more often than less readily available ones according to the Principle of Least Effort [28,29]; and 3) symbolism, *e*.*g*. mango and sandalwood are regarded as sacred by the Aryans in India because of their sacred association and often used on special occasions such as cremation and sacrifice[30], and in fact are still used by Buddhists in Bangladesh when building cremation pyres [31].

Alpine desert vegetation dominated by *Ceratoides compacta* and *Artemisia rhodantha* is found in the dry, high-altitude Tashkurgan area [32]. Alpine cushion-like vegetation and alpine desert steppe are also present. In flood plains, *Caprifoliaceae* and *Rosaceae* shrubs are common, while *Hippophaer hamnoides* L., *Salix dasyclados* and *Salix carmanica* are the dominant species in river valleys. With the increase of altitude, fruticous desert dominated by *ephedra*, alpine desert consist of *Seriphidium* and alpine steppe composed of *stipa* are in belt shaped distribution. Above the alpine steppe, alpine swamp and meadow can be found in small area [33]. Natural forest in mountainous regions is composed principally of *Sabina chinensis*, *Picea schrenkiana* and *Betula* [33,34].

At the Ji’erzankale Necropolis, the timber came from taxa including *Sabina* sp., *Populus* sp., *Betula* sp., *Salix* sp., *Lonicera* sp., *Elaeagnus angustifolia* and *Fraxinus syriaca*, mainly the common woody plants in the Pamir region. Local residents appear to have chosen timber based upon its immediate availability in the harsh environment of this dry, high-altitude plateau, and to have adapted their needs to the particular characteristics of these local woods rather than ranging more widely in order to find other timber.

Properties such as density, intensity, rigidity, inflammability, and corrosion resistance usually represent significant criteria in wood selection. In general, the higher the density, the higher pyroconductivity, which means that denser wood, is more difficult to ignite (requiring a greater specific heat and longer ignition time) than lighter timber and *vice versa* [35–37]. *Populus* has a relatively low density, and is thus easy to ignite [18]. *Salix* is characterized by abundant and coriaceous straight-grained branches with high impact resistance and considerable elasticity. Being hard to crack after drying, it is suitable as a material for stick-shaped objects such as sports mallets and shoulder poles [18]. *Sabina* has a fine structure, oblique texture and low tensile strength, and releases a full-bodied turpentine perfume odour upon burning [18,38]. *Betula* possesses a straight-grained, homogeneous structure, relatively high rigidity and intensity, a high density, and low inflammability [18]. *Fraxinus syriaca* has a beautiful grain and is easy to process. *Lonicera* is notable for its high rigidity and numerous, straight branches.

At the Ji’erzankale Necropolis, relatively inflammable *Populus* was frequently selected for hearth board and hand drill manufacture. The three wooden sticks from the M14 tomb are all *Salix*. This wood was most likely used for its abundance of tough branches, which do not usually deform after drying. Combined with its durability over time, this property makes *Salix* excellent for stick manufacture. *Betula*, *Populus* and *Sabina* were selected for the 11 fire altars. *Betula* may have been preferred for the manufacture of wooden objects since it was the commonest species in the Tashkurgan region. *Populus* can be ignited easily and so is an obvious choice for the manufacture of objects intended to be burned. As for *Sabina* used for making fire altar the smell released by this wood as it burns makes it useful during particular rites. In New Zealand and Melanesia, sandalwood, mango, wood-apple and juniper, which are all perfumed, were used to obscure the smell of burning corpses [39,40]. *Sabina* may well have been chosen, therefore, for its tendency to produce a full-bodied turpentine smell.

Most (19) of the 30 wooden plates excavated at the site were made from *Betula*. The high rigidity and density of this wood, as well as its homogeneous texture, all make it suitable for the manufacture of objects for holding produce.

Ancient peoples knew that arrow shafts needed to be made from wood with a high rigidity. One famous example was brought to light by one German couple who found the Ötzi ‘Iceman’ (ca. 5300 yr BP) in Italy. For the shafts of his arrows, the iceman chose the wood of the common wayfaring tree (*Viburnum lantana*), whose rigidity makes it superior for that purpose to any other wood type indigenous to the region. Only one of his 14 arrows did he make use of a second-best material, namely the wood of the cornel tree (*Cornus* sp.)[41]. The eight arrow shafts found at the Ji’erzankale Necropolis were all of *Lonicera*, which has straight branches and high rigidity. The abundance of branches with an even diameter of 1–2 cm makes it particularly easy to produce arrow shafts by simply wiping off the rhytidome and soft bark. *Lonicera* is therefore an ideal material and was widely used throughout the Pamir for making arrow shafts.

The shoo konghou originated in Egypt (where it was called the harp) and West Asia (the Assyrians called it Cank). Painted wooden figurines playing harps were buried in Pharaoh’s tomb, 3000 BC [42]. Six konghous were found in Zagunluk and Yanghai graveyard, Xinjiang, in 1996 and 2004 [43,44]. The resonating chambers of these six instruments were all made from *Populus euphratica*. The two konghous recovered from the Ji’erzankale Necropolis were made from *Populus* and *Fraxinus syriaca*. One harp was from *Fraxinus syriaca*. *Populus* was probably selected for the purpose of making instruments because of its high availability, whereas the beautiful grain of *Fraxinus syriaca* made it highly suitable for musical instrument manufacture. The konghou was transmitted from West Asia to the Western Regions and then to Central China. Evidence from the Ji’erzankale Necropolis showed that this distinctive musical instrument had been transported as far as western China by 2500 years ago.

Among the seven types of timber found in the Ji’erzankale Necropolis, *Sabina* and *Betula* occur in forested mountain areas, while *Populus*, *Salix*, *Elaeagnus angustifolia* and *Lonicera* are common in river valleys. Therefore, we can infer that *Sabina*, *Betula*, *Populus*, *Salix*, *Elaeagnus angustifolia*, and *Lonicera* were mainly among the most common species of wood in the Tashkurgan area in 2500 yr BP. *Fraxinus syriaca* which can be seen in Afghanistan and Pakistan today and making some exotic objects like shoo konghou and harp, may grew in Pamir region at that time since it is difficult to distinguish whether these exotic musical instruments were acquired through commerce exchange. An analysis of the wooden objects found in 25 tombs shows that the people of the Pamir were able to utilize the natural resources at hand in a way which allowed them to adapt their customs and practices to a harsh, high-altitude environment. They took full advantage of the available species and made the objects they required by exploiting the characteristics of the timbers to which they had access. Inflammable *Populus* was selected for tools intended to be burned, and *Betula* with its high intensity and rigidity was used for the manufacture of wooden plates. Perfumed *Sabina* was preferred for fire altars, and high-rigidity *Lonicera* was the first choice for arrow shafts. *Salix*, with its high impact resistance and considerable elasticity, was used for making sticks and crutches. *Fraxinus syriaca*, which has a beautiful grain, was often chosen for crafting musical instruments.

Besides an integrated analysis of wood usage, some valuable information of burial custom was also revealed by fire altars. They were placed in this cemetery along with the dead as burial objects with 15 stones inside. The diameter of central burning hole is less than 15 cm (one object is only 7 cm), and all of them were made from inflammable timber rather than some fireproof materials like stone, crockery, or bronze (all were found in this cemetery). In addition, *Sabina* is rare in Tashkurgan region as only 4 *Sabina* objects were found in our total 70 samples and among the four *Sabina*, three were used for fire altar making, one for hearth board crafting. The small size and conscious selection of *Sabina* wood probably gave an implication that they were not used as ordinary practical brazier for warming purpose but some extraordinary appliances with symbolic meaning. In conclusion, fire played an important role in funerary and veneration of fire, presumably, was prevalent among the peoples lived in Pamir region 2500 years ago.

In the light of current archaeological evidence from central Asia, fire veneration was prevalent among the Scythians and Persians. They regarded fire as sacred and offered sacrifices to it [6,45]. Veneration of fire was clearly also practiced by the Zoroastrian [46,47]. For them, fire is sacred and regarded as a means to communicate with the divinity Ātar who lived among men as their servant and master [48–51]. Therefore, fire was present at their religious ceremonies and sacrificial fire objects were buried with the dead [51–54]. According to the integrated study of excavated 50 tombs, secondary burial of corpse after a transitory exposure in air and fire altar showing veneration of fire revealed that the influence of Zoroastrianism may have reached the Tashkurgan region where was regarded as an important culture crossroad between West and East.

## Conclusion

The wood used to manufacture various objects found in the Ji’erzankale Necropolis came from seven plant taxa: *Betula* sp., *Populus* sp., *Salix* sp., *Sabina* sp., *Lonicera* sp., *Fraxinus syriaca* and *Elaeagnus angustifolia*. These represent the principal locally occurring trees and shrubs. On the one hand, people largely used the widely distributed *Betula* and *Populus*. On the other hand, they made more selective and specific use of *Populus* for hand drills and hearth boards, *Betula* for wooden plates, *Sabina* for fire altars, *Lonicera* for arrow shafts, *Salix* for wooden sticks and crutches, and *Fraxinus syriaca* for musical instruments. Conscious selection of given wood types for particular purposes indicated that the early residents of the Pamir Plateau made full use of the available timber and were adept at crafting objects out of wood in a manner that took into account the properties of specific types of timber. This highlights their sophistication and ability to adapt to the local environment. Fire veneration revealed by small-sized and rounded fire altars made from aromatic *Sabina*, as well as the secondary burial after a short-time exposure implied Tashkurgan region which was characteristic of culture transmission between East and West, may have been influenced by Zoroastrianism 2500 years ago.

## Supporting Information

S1 Table Full details of the wood objects from Ji’erzankale Necropolis. (XLSX)

## References

[1] Liu XT. Toward the cultural communication between East and West during the Stone Age. Journal of Xinjiang Normal University (Social Sciences). 2012; 33:47–56.

[2] Hou YM. Shuidonggou: A vane of intercommunication between the East and the West? Discussion about small tool culture in north China and a hypothesis of the Lithic Road. Quaternary Sciences. 2005; 25: 750–761.

[3] Wu XH. Archaeological excavation of Ji'erzankale necropolis in Tashkurghan, Xinjiang, 2013. The Western Regions Studies. 2014; 4: 36–46.

[4] Li JX. Religion research in Silk Road Urumqi: Xinjiang People’s Publishing House; 2009.

[5] Wu Y. Bronze Age cultural of Xiabandi tomb in Kashgar, Xinjiang. The Western Regions Studies. 2012; 4: 36–46.

[6] Wang ZL. General history of central Asia Urumqi: Xinjiang People’s Publishing House; 2007.

[7] Stausberg M. On the state and prospects of the study of Zoroastrianism. Numen. 2008; 55: 561–600.

[8] Li XQ, Sun N, Dodson J, Zhou XY. Human activity and its impact on the landscape at the Xishanping site in the western Loess Plateau during 4800–4300 cal yr BP based on the fossil charcoal record. Journal of Archaeological Science. 2012; 39: 3141–3147.

[9] Sun N, Li XQ, Dodson J, Zhou XY, Zhao KL, Yang Q. Plant diversity of the Tianshui Basin in the western Loess Plateau during the mid-Holocene–Charcoal records from archaeological sites. Quaternary International. 2013; 308–309: 27–35.

[10] Wang SZ. Brief history of wood remains in archaeological sites. Southern Heritage. 2011; 1: 156–162.

[11] Carcaillet C. Charred particles analyses. Encyclopedia of Quaternary Science. 2007; 1582–1593.

[12] McGinnes E Jr, Szopa P, Phelps J. Use of scanning electron microscopy in studies of wood charcoal formation Chicago: Inst; 1974.

[13] Figueiral I, Carcaillet C. A review of Late Pleistocene and Holocene biogeography of highland Mediterranean pines (Pinus type sylvestris) in Portugal, based on wood charcoal. Quaternary Science Reviews. 2005; 24: 2466–2476.

[14] Scott AC, Damblon F. Charcoal: Taphonomy and significance in geology, botany and archaeology. Palaeogeography, Palaeoclimatology, Palaeoecology. 2010; 291: 1–10.

[15] Scott AC. Charcoal recognition, taphonomy and uses in palaeoenvironmental analysis. Palaeogeography, Palaeoclimatology, Palaeoecology. 2010; 291: 11–39.

[16] Yang SP, Xu HY, Yan P. Floristic Elements of Seed Plants in the Pamirs Region of China. Chinese Bulletin of Botany. 2007; 24: 597–602.

[17] Cao SQ. Softening method and bending technique. Technology. 2006; 26–28.

[18] Cheng JQ, Yang JJ, Liu P. Atlas of Wood in China. Beijing: China Forestry Publishing House; 1992.

[19] Zhou Q, Jiang XM. Wood anatomy and ultrastructure of gymnospermous woods in China Beijing: China Forestry Publishing House; 1994.

[20] Fahn A, Werker E. Wood anatomy and identification of trees and shrubs from Israel and adjacent regions Jerusalem: The Israel Academy of Sciences and Humanities; 1986.

[21] Xu F. Anatomical figures for wood identification Beijing: Chemical industry press; 2008.

[22] Deforce K, Haneca K. Ashes to ashes. Fuelwood selection in Roman cremation rituals in northern Gaul. Journal of Archaeological Science. 2012; 39: 1338–1348.

[23] Deforce K, Haneca K. Tree-ring analysis of archaeological charcoal as a tool to identify past woodland management: The case from a 14th century site from Oudenaarde (Belgium). Quaternary International. 2014; 1–11.

[24] Pessenda LCR, Gouveia SEM, Aravena R, Boulet R, Valencia EPE. Holocene fire and vegetation changes in southeastern Brazil as deduced from fossil charcoal and soil carbon isotopes. Quaternary International. 2004; 114: 35–43.

[25] Li XQ, Sun N, Dodson J, Zhou XY, Zhao KL. Vegetation characteristics in the western Loess plateau between 5200 and 4300 cal.B.P. based on fossil charcoal records. Veget Hist Archaeobot. 2013; 22: 61–70.

[26] Cope MJ, Chaloner WG. Fossil Charcoal as Evidence of Past Atmospheric Composition. Nature. 1980; 283: 647–649.

[27] Moskal-del Hoyo M. The use of wood in funerary pyres: random gathering or special selection of species? Case study of three necropolises from Poland. Journal of Archaeological Science. 2012; 39: 3386–3395.

[28] Prior J, Price Williams D. An investigation of climatic change in the holocene epoch using archaeological charcoal from Swaziland, Southern Africa. Journal of Archaeological Science. 1985; 12: 457–475.

[29] Shackleton CM, Prins F. Charcoal analysis and the “Principle of least effort”—A conceptual model. Journal of Archaeological Science. 1992; 19: 631–637.

[30] Upadhyaya KD. Indian botanical folk lore. Asian Folk lore Studies. 1964; 23: 15–34.

[31] Barua DK. Funeral rituals of Buddhist in Bangladesh. Journal of Indian and Buddhist Studies. 2003; 51: 1017–1021.

[32] Zhang XS. China's vegetation and its geographical pattern Beijing: Geological Publishing House; 2007.

[33] Xinjiang integrated Expedition Team and Institute of Botany CASS. Vegetation and its utilization in Xinjiang Beijing: Science Press; 1978.

[34] Guo K, Zheng D. Regional differentiation of vegetation on the west Kunlun, the west Karakorum, and the north- west Himalaya and the implication for the ecological environment. Acta Phytoecologica Sinica. 2002; 26(1): 17–22.

[35] Hollman JR. Ignition characteristics of plastics and rubber, Ph.D. Dissertation. Norman: Univ. of Oklahoma; 1971.

[36] Lawson D, Simms D. The ignition of wood by radiation. British Journal of Applied Physics. 1952; 3: 288–292.

[37] Wu YZ, Toshiro H. Study on the Burning Behaviour of Plantation Wood. Scientia Silvae Sinicae. 2004; 40: 131–136.

[38] Editorial Board of “flora of china” CAS. Flora of China. Beijing: Science Press; 2004.

[39] Malanggan KS. Art, Memory and Sacrifice. Berg: Oxford; 2002.

[40] Williams HMR. Death warmed up: the agency of bodies and bones in Early Anglo-Saxon cremation rites. Journal of Material Culture. 2004; 9: 263–291.

[41] Bortenschlager S, Oeggl K. The Iceman and his Natural Environment. Wien: Springer-Verlag; 2000.

[42] Lin MC. Fifteen lectures on archaeology of the Silk Roads by Lin Meicun Beijing: Peking University Press; 2006.

[43] Academia Turfanica, Xinjiang and Xinjiang institute of Culture Relics and Archaeology. Excavation on the Yanghai cemetery in Shanshan (Pian) country, Xinjiang. Acta Archaeologica Sinica. 2011; 1: 99–150.

[44] He ZL. Shape origin of the relic harp of Uigur area. Musicology in China. 2006; 1: 43–52.

[45] Herodotus. History (Chinese Translation, 1985 version). Beijing: Commercial Press; 1985.

[46] Han X. Exotic religion in Tang dynasty and civilization of central Asia. Journal of Shanghai Normal University. 2006; 35: 57–62.

[47] Mary B. On the orthodoxy of Sasanian Zoroastrianism London: Cambridge University Press; 2013.

[48] Li JX. The transmission of Zoroastrianism in Xinjiang and its regional character. The Western Regions Studies. 2007; 1: 81–87.

[49] Zhou JB. Zoroastrianism civilization in the Western Region. North West Ethno-national Studies. 1991; 1: 115–125.

[50] Mary B. On the Zoroastrian temple cult of fire. Journal of the American Oriental Society. 1975; 95(3): 454–465.

[51] Mary B. Zoroastrians: Their religious beliefs and practices London: Routledge & Kegan Paul Ltd; 1979.

[52] Lin MC. The first transmission of Zoroastrianism in China from archaeological evidence. The Western Regions Studies. 1996; 4: 54–60.

[53] Wang BH. Brief report on the excavation of Alagou tomb in Xinjiang. Cultural Relics. 1981; 1:18–23.

[54] Yang JX. Silk Road. Lanzhou: Gansu People’s Publishing House; 1988.

